# A Micro Bubble Structure Based Fabry–Perot Optical Fiber Strain Sensor with High Sensitivity and Low-Cost Characteristics

**DOI:** 10.3390/s17030555

**Published:** 2017-03-09

**Authors:** Lu Yan, Zhiguo Gui, Guanjun Wang, Yongquan An, Jinyu Gu, Meiqin Zhang, Xinglin Liu, Zhibin Wang, Gao Wang, Pinggang Jia

**Affiliations:** 1 School of Information and Communication Engineering, North University of China, Taiyuan 030051, China; S1505018@st.nuc.edu.cn (L.Y.); guizhiguo@nuc.edu.cn (Z.G.); S1505032@st.nuc.edu.cn (J.G.); S1505016@st.nuc.edu.cn (M.Z.); S1505033@st.nuc.edu.cn (X.L.); wangzhibin@nuc.edu.cn (Z.W.); wanggao@nuc.edu.cn (G.W.); 2 Engineering Technology Research Center of Shanxi Province for Opto-electric Information and Instrument, Taiyuan 030051, China; 3 School of Instrument and Electronics, North University of China, Taiyuan 030051, China; pgjia@nuc.edu.cn

**Keywords:** optical fiber sensor, Fabry–Perot, strain measurement

## Abstract

A high-sensitivity, low-cost, ultrathin, hollow fiber micro bubble structure was proposed; such a bubble can be used to develop a high-sensitivity strain sensor based on a Fabry–Perot interferometer (FPI). The micro bubble is fabricated at the fiber tip by splicing a glass tube to a single mode fiber (SMF) and then the glass tube is filled with gas in order to expand and form a micro bubble. The sensitivity of the strain sensor with a cavity length of about 155 μm and a bubble wall thickness of about 6 μm was measured to be up to 8.14 pm/με.

## 1. Introduction

The micro fiber Fabry–Perot interferometer (FPI) sensor with the advantages of compact structure [1], anti-electromagnetic interference [2], and high sensitivity [3] has taken the leading role in a large number of sensing applications such as strain, high sensitive temperature, high sensitive pressure and so on [4,5,6]. It has been widely used in biomedical, non-destructive health monitoring and other fields; among them, the Fabry–Perot (FP) can be effectively used in the optical fiber tip for a limited space environment. This is a powerful function in harsh natural and chemical environments [7,8].

At present, optical fiber micro air bubbles can be manufactured by various technologies, such as the use of hydrofluoric acid etching, due to the core material of doped germanium and pure silica materials with different corrosion rates of oxygen fluorine acid; after a period of time after reaction, a groove structure will appear on the endface of the optical fiber, and melt the two grooves together, which can achieve an optical fiber FP interference structure [9,10]. Jiang Xiaogang used the chemical etching method to make a groove in the tip of a multimode fiber (MMF); the etched fiber would be put into a fusion splicer and then a micro-cavity would be created in the tip of the optical fiber by using arc discharge at the fiber end [11]. However, this kind of structure reduces the device’s characteristics because the corrosion process makes a certain roughness in the concave holes. Due to the discharge current and parameters factor, the bubble will be formed within the fiber in the fusing splicer.

Recently, a new method was introduced by the Li group [12] and the Villatoro group [13]; it was fusion splicing together a section of conventional single mode fiber (SMF) and a section of hollow core or solid core photonic crystal fiber (PCF) to form a micro bubble. Their fabricated FPIs have a high strain sensitivity: 3.36 pm/με and 2.7 pm/με for the FPI fabricated by the Li group and Villatoro group respectively. Therefore, they are suitable for strain measurement. However, the wall thickness of the micro bubble structure made by PCF is random and uncontrollable.

Ma Jun et al. from Hong Kong Polytechnic University fabricated the sensor by splicing a silica capillary to a SMF and then fusing (heating/melting) the capillary to form a microsphere with an internal air-cavity [14]. The FPI strain sensor of the air bubble was fabricated by two standard SMF and formed by arc fusion splicing; Chongqing University’s experimental results showed the strain sensitivity to be ~4 pm/με [15]. Shenzhen University reported a high-sensitivity of up to 6.0 pm/με [16], and then they improved the technique to create a rectangular air bubble based FPI with a cavity length of about 61 μm; the wavelength of 1550 nm exhibits a high strain sensitivity of 43.0 pm/με [17].

In view of the above situation, this paper demonstrates an improved simple encapsulation method for the preparation of a high-sensitivity, low-cost, ultrathin, hollow fiber micro bubble structure, which is realized by the method of multiple weak discharges and slow release pressure at the end of the optical fiber. In the end, a micro bubble wall with a thickness of about 3~8 μm was prepared. The strain sensitivity analysis uses the FP interference technique. The experimental results show that the micro bubble has the strain sensitivity of 8.14 pm/με. Finally, the results which combined with the ANSYS software and the experiment are explained.

## 2. Sensor Fabrication

**Figure 1:**
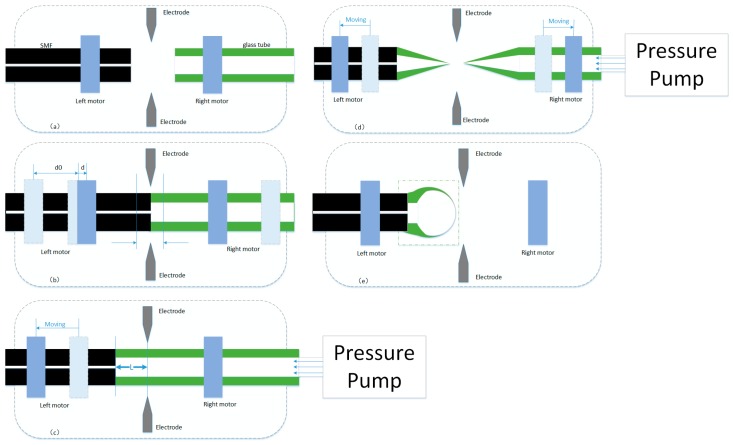
Schematic diagrams of the fabrication process of in-fiber FPI based on an air bubble. (**a**) SMF and glass tube are placed in the fusion splicer; (**b**) Splice a glass tube to a SMF; (**c**) heat and melt the glass tube to form an air bubble (L: Distance from electrode to the SMF end); (**d**) The glass tube is separated into two parts by applying pressure to the glass tube section several times at the moment of arc discharges; (**e**) a sketch showing the fiber-tip micro bubble.

Figure 1 shows that the fabrication process of an ultrathin, hollow fiber micro bubble structure uses the pressure-assisted arc discharge technology, which involves five steps. In step 1, as shown in Figure 1a, SMF with an outer diameter D = 125 µm and an inner diameter d = 8 µm, made of silica material, and a glass tube with an outer diameter D = 125 µm and an inner diameter d = 75 µm, are placed in the left and right motor of the fusion splicer (Fijikura FSM 60S, Fijikura, Tokyo, Japan). In step 2, as shown in Figure 1b, the optical fiber and glass tube discharge are welded by the driving motor. In step 3, as shown in Figure 1c, the left motor is driven so that the position of the splicer’s electrodes offsets the splice joint by L. In step 4, as shown in Figure 1d, the pressure pump (ConST162, ConST, Beijing, China) is connected to fill the inner wall of the sealed glass tube with an absolute pressure of about 120 KPa. The glass tube is separated into two parts by applying pressure to the glass tube section several times at the moment of arc discharges. The temperature reaches the softening/melting point of glass at a high discharge current (~20 mA). In step 5, as shown in Figure 1e, melting discharge is continued in the end of the glass tube to form a bubble structure, which is shaped by the air trapped in the glass tube during the discharge. The bubble wall is relatively thick at the beginning; after a few discharges, the micro bubble region will expand again; correspondingly, the bubble wall also becomes thinner than before. Finally, the thickness of the micro bubble wall can reach several microns or even micron level, and is relatively uniform.

**Figure 2:**
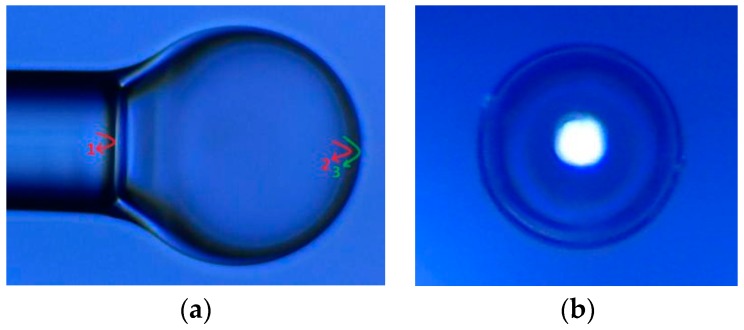
(**a**) Microscope image of the micro bubble; (**b**) Top view of the micro bubble.

Figure 2 is the picture of the preparation of the micro bubble structure, under the 20X microscope. From the microscope images, the micro bubble length and the bubble wall thickness are estimated to be 155 μm and 6 μm respectively. The uniform thickness of the wall is very important for the fabrication of the ultrathin micro bubble structure by controlling the arc discharge and motion parameters. Here, we choose the discharge time of 300 ms and the discharge intensity of −5 bit. Because of the uneven thickness of the bubble structure, it is easy to rupture at the end of the discharge process. In the same discharge, the thinner area is easier to soften and expand; after reaching a certain limit, the bubble will burst.

**Figure 3:**
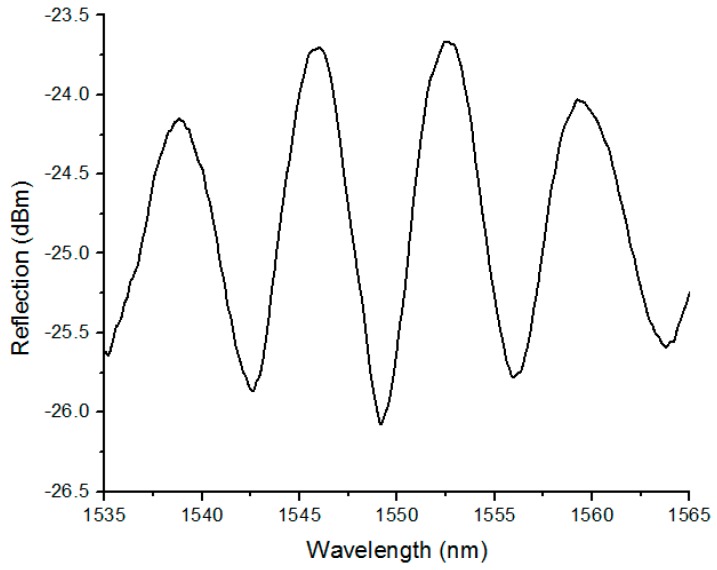
Measured reflection spectrum of the micro bubble sensor shown in Figure 2a.

Three reflected waves are found when light is shone into SMF, as shown in Figure 2a; No. 1 is from the end of the SMF, and No. 2 and No. 3 are from the inner and outer surfaces of the bubble wall respectively. Figure 3 shows the measured reflection spectrum of the micro bubble sensor shown in Figure 2a. Since the bubble wall is thin, the edges displayed in Figure 3 may be approximately considered as the result of the interference of two-waves; the optical path difference is twice as much as the cavity length. If a two-wave interference model is used, the fringe spacing Δλ can be calculated by Δλ = λ^2^/2*nd*, where *n* (≈1) is the refractive index of air and *d* is the cavity length. The bubble, as shown in Figure 2a, Δλ was calculated to be ~7.5 nm, which agrees with the value of 7 nm measured from Figure 3 at wavelength 1550 nm.

## 3. Analysis of the Mechanical Properties of Micro Bubbles

How to apply the load to the sensing probe accurately and effectively is the main problem of the system. According to the existing laboratory conditions, we made polydimethylsiloxane (PDMS) diaphragms, fixed the optical fiber micro bubble in the middle of the two PDMS diaphragms and placed them on the electronic balance.

With different weight put on the PDMS diaphragms, the stress applied to the micro bubble can be decomposed into $\sigma_{r}$, $\sigma_{\theta }$ and $\sigma_{s}$—three directions in the coordinate system. The simple $\sigma_{s}$ is the role of axial stress; $\sigma_{r}$ and $\sigma_{\theta }$ are transverse stress; the three existing simultaneously show the effect of body stress.

The general form of Hooke’s theorem can be expressed by the following formula:

(1) $$\sigma_{i}=C_{ij}\varepsilon_{j}(i,j=1,2,3,4,5,6)$$

where $\sigma_{i}$ is the stress tensor; $C_{ij}$ is the elastic modulus; ε is the strain tensor.

For isotropic media, the $C_{ij}$ can be simplified because of the symmetry of the material; the constants *λ* and *μ* are used to represent the elastic modulus:

(2) $$\left[\begin{array}{c} \sigma_{1}\\ \sigma_{2}\\ \sigma_{3}\\ \sigma_{4}\\ \sigma_{5}\\ \sigma_{6} \end{array}\right]=\left[\begin{array}{cccccc} \lambda +2\mu & \lambda & \lambda & 0 & 0 & 0\\ \lambda & \lambda +2\mu & \lambda & 0 & 0 & 0\\ \lambda & \lambda & \lambda +2\mu & 0 & 0 & 0\\ 0 & 0 & 0 & \mu & 0 & 0\\ 0 & 0 & 0 & 0 & \mu & 0\\ 0 & 0 & 0 & 0 & 0 & \mu \end{array}\right]•\left[\begin{array}{c} \varepsilon_{1}\\ \varepsilon_{2}\\ \varepsilon_{3}\\ \varepsilon_{4}\\ \varepsilon_{5}\\ \varepsilon_{6} \end{array}\right]$$

The Lame constants *λ* and *μ*, which can be expressed by the material elastic modulus *E* and Poisson’s ratio *ν*:

(3) $$\lambda =\frac{\nu E}{\left(1+\nu )(1-2\nu \right)}$$

(4) $$\mu =\frac{E}{2(1+\nu )}$$

This formula is the general form of Hooke’s theorem in homogeneous medium.

Here, the fiber micro bubble is mainly affected by the transverse stress; the uniform transverse stress *P* is applied to the optical fiber along each of the radial directions; the corresponding internal stress state of the optical fiber is $\sigma_{rr}=\sigma_{\theta \theta }=-P$; and there is no shear strain in $\sigma_{ss}=0$, based on the generalized Hooke theorem in the context of the fiber strain tensor:

(5) $$\left[\begin{array}{c} \sigma_{rr}\\ \sigma_{\theta \theta }\\ \sigma_{ss} \end{array}\right]=\left[\begin{array}{c} -(1-\nu )\frac{P}{E}\\ -(1-\nu )\frac{P}{E}\\ 2\nu \frac{P}{E} \end{array}\right]$$

**Figure 4:**
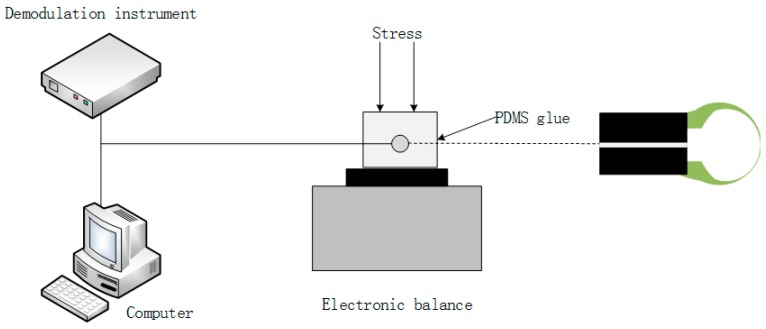
Setup for measuring the reflection spectrum of the micro bubble strain sensor.

The schematic diagram of the experimental test system is shown in Figure 4.

**Figure 5:**
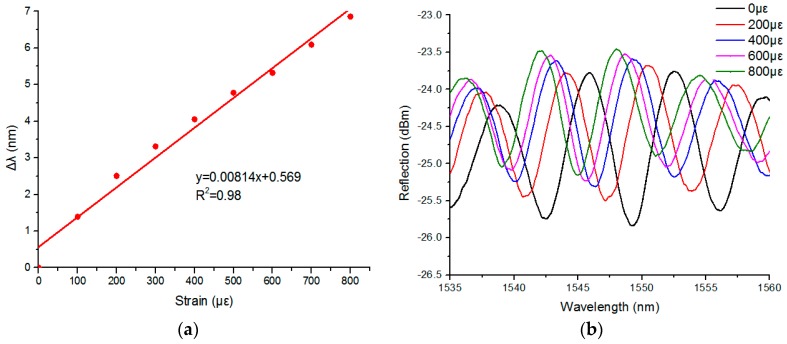
(**a**) Wavelength shift of the interference fringe around 1555 nm as a function of tensile strain applied to the micro bubble; (**b**) Reflection spectrum evolution of the air bubble, while the tensile strain increases from 0 to 800 με.

When the demodulation has an inner light source, and provides an effective wavelength range of 1525 nm to 1570 nm, then the reflection spectrum was displayed on the computer. The strain characteristics of the sensing head were fixed in the middle of the two PDMS diaphragms and tested under a constant temperature (~18 °C), then they were placed on an electronic balance. The reflection spectrum of the sensor was recorded without the weight. In our experiment, Figure 5a shows that a linear fitting to the experimental data gives a wavelength–strain sensitivity of 8.14 pm/με, and a high coefficient of determination value of R^2^ (0.98); R^2^ demonstrates that the linearity of the spectrum dip strain response is excellent. Figure 5b shows that the measured transmission spectra were applied to strains of 0 με to 800 με in steps of 100 με. When the applied transverse stress was gradually increased, the interference spectrum shifted to the short wave direction, and a red-shift of the reflection spectrum was observed since the micro-cavity elongates laterally. It was found that the reflection spectrum is no longer moving when the stress reaches a certain value.

## 4. Numerical Analysis

**Figure 6:**
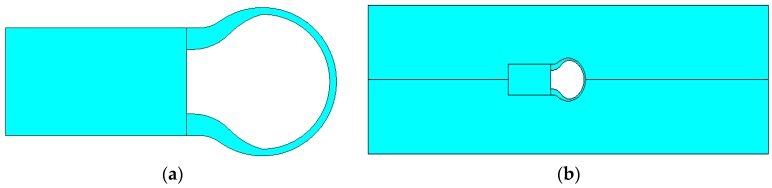
ANSYS simulation diagram. (**a**) The model of the micro bubble; (**b**) The micro bubble is fixed in the middle of two PDMS diaphragms.

**Figure 7:**
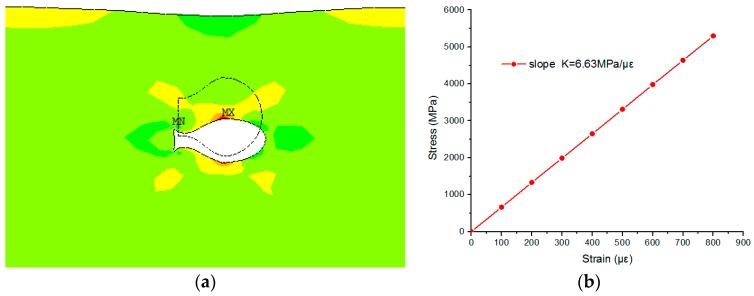
(**a**) ANSYS stress contours of the micro bubble. MX shows the maximum displacement variation, whereas MN is the minimum displacement variation; (**b**) Calculated stress of air bubble versus the applied strain.

In order to study the stress deformation and the deformation of optical fiber micro bubbles under an applied tensile strain, simulation models were established by use of ANSYS software, and the measured size of the air bubble was illustrated in Figure 2a. The Young’s modulus and Poisson’s ratio of optical fiber and PDMS are 73 GPa, 0.17 and 1.2 GPa, 0.48, respectively. Figure 6a is a model of the optical fiber micro bubble, and Figure 6b shows that a micro bubble is fixed in the middle of the two PDMS diaphragms. Figure 7a illustrates the two-dimensional stress contours of the micro bubble with a tensile strain of 100 με, which indicates the calculated stress distribution in different parts of the micro bubble. While the applied tensile strain increases, as shown in Figure 7b, the top of the micro bubble is subjected to the maximum stress—the calculated stress at the micro bubbles—linearly, with a slope of 6.63 MPa/με.

## 5. Conclusions

This paper summarizes the existing fiber micro bubbles technology. We demonstrate a method with multiple, pressure-assisted arc discharges for preparing a high-sensitivity, low-cost, ultrathin, hollow fiber micro bubble structure; after optimization of the related parameters, the thickness of the micro bubble wall can reach 3~8 microns, achieve good uniformity, and the thickness of the wall could be controlled. Such an in-fiber micro bubble can be used to develop a high-sensitivity strain sensor based on FP interference. The sensitivity of the strain sensor is up to 8.14 pm/με. Finally, the experimental results are explained by the simulation of ANSYS software.
