# Correction: Yan, L., et al. A Micro Bubble Structure Based Fabry–Perot Optical Fiber Strain Sensor with High Sensitivity and Low-Cost Characteristics *Sensors*, 2017, *17*, 555.

**DOI:** 10.3390/s18093102

**Published:** 2018-09-14

**Authors:** Lu Yan, Zhiguo Gui, Guanjun Wang, Yongquan An, Jinyu Gu, Meiqin Zhang, Xinglin Liu, Zhibin Wang, Gao Wang, Pinggang Jia

**Affiliations:** 1 School of Information and Communication Engineering, North University of China, Taiyuan 030051, China; S1505018@st.nuc.edu.cn (L.Y.); guizhiguo@nuc.edu.cn(Z.G.); S1505032@st.nuc.edu.cn (J.G.); S1505016@st.nuc.edu.cn (M.Z.); S1505033@st.nuc.edu.cn (X.L.); wangzhibin@nuc.edu.cn (Z.W.); wanggao@nuc.edu.cn (G.W.); 2 Engineering Technology Research Center of Shanxi Province for Opto-Electric Information and Instrument, Taiyuan 030051, China; 3 School of Instrument and Electronics, North University of China, Taiyuan 030051, China; pgjia@nuc.edu.cn

**Keywords:** optical fiber sensor, Fabry–Perot, strain measurement

## Abstract

An correction is presented to correct Figure 5a in [*Sensors*, **2017**, *17*, 555].

The authors of [1] which to replace Figure 5a with the following:

In this corrected figure, the data was fitted in exponential, but not linear function [1]. The authors regret this mistake. Thus, the fitted function can be depicted as Δ*λ* = −11.39751 × exp(−*δ*/939.38195) + 11.54492. The corresponding sensitivity *S* could be calculated by differentiating the upper formula, which is *S* = 0.012133 × exp(−*δ*/939.38195), and the maximum strain sensitivity of the proposed microbubble is 12.133 pm/με.

The changes do not affect the scientific results. The manuscript will be updated and the original will remain online on the article webpage, with a reference to this Correction.

## Figures and Tables

**Figure 5:**
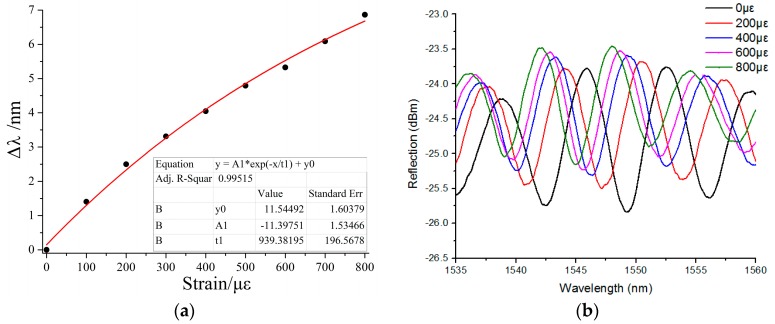
Strain sensitivity characteristics of proposed microbubble. (**a**) Wavelength shift of the interference fringe around 1555 nm as a function of tensile strain applied to the micro bubble; (**b**) Calculated sensitivity.
